# Supplementary material for: Maternal Gut Dysbiosis Alters Offspring Microbiota and Social Interactions
Source: Microorganisms. 2021 Aug 15;9(8):1742. doi: 10.3390/microorganisms9081742 (PMC8401725; doi:10.3390/microorganisms9081742)
Supplement: Supplementary file 1 [file microorganisms-09-01742-s001.zip › microorganisms-1350038-supplementary.pdf]

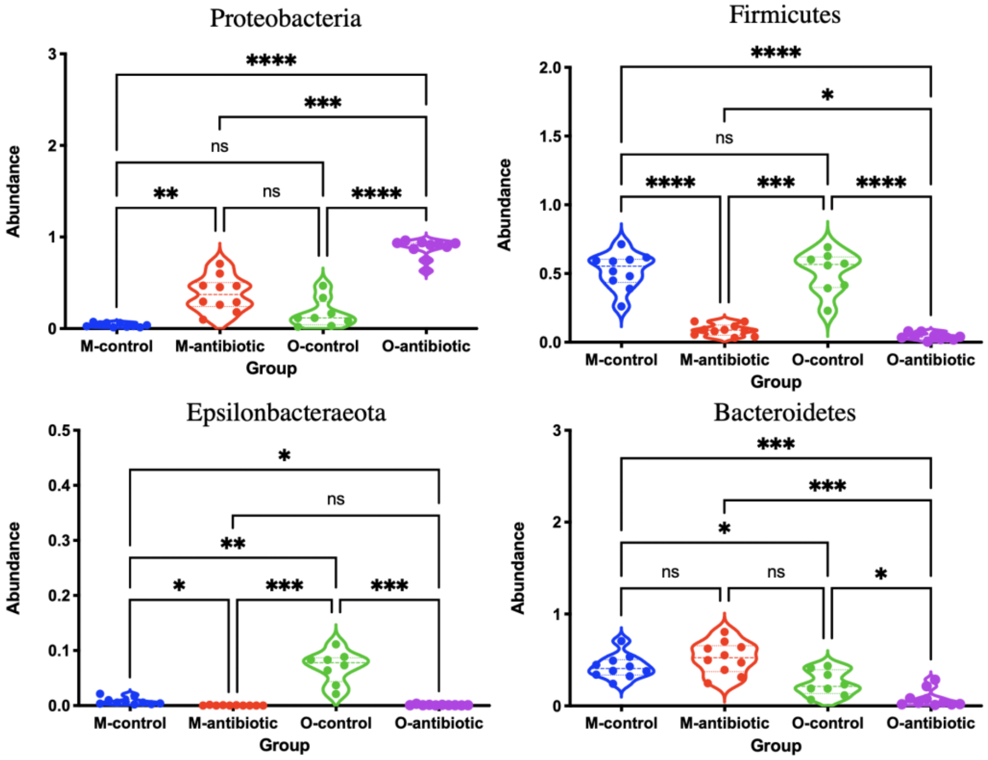

Figure S1. Relative abundance of four phyla that were significantly different in the gut microbiota in the maternal and offspring samples. M-antibiotics and M-control stand for antibiotics-treated mothers and control mothers, respectively, while M-antibiotics and M-control represent offspring born to antibiotics-treated mothers and control mothers, respectively. Statistical significance: *, P < 0.05; **, P < 0.01; ***, P < 0.001; ns, not significant. .

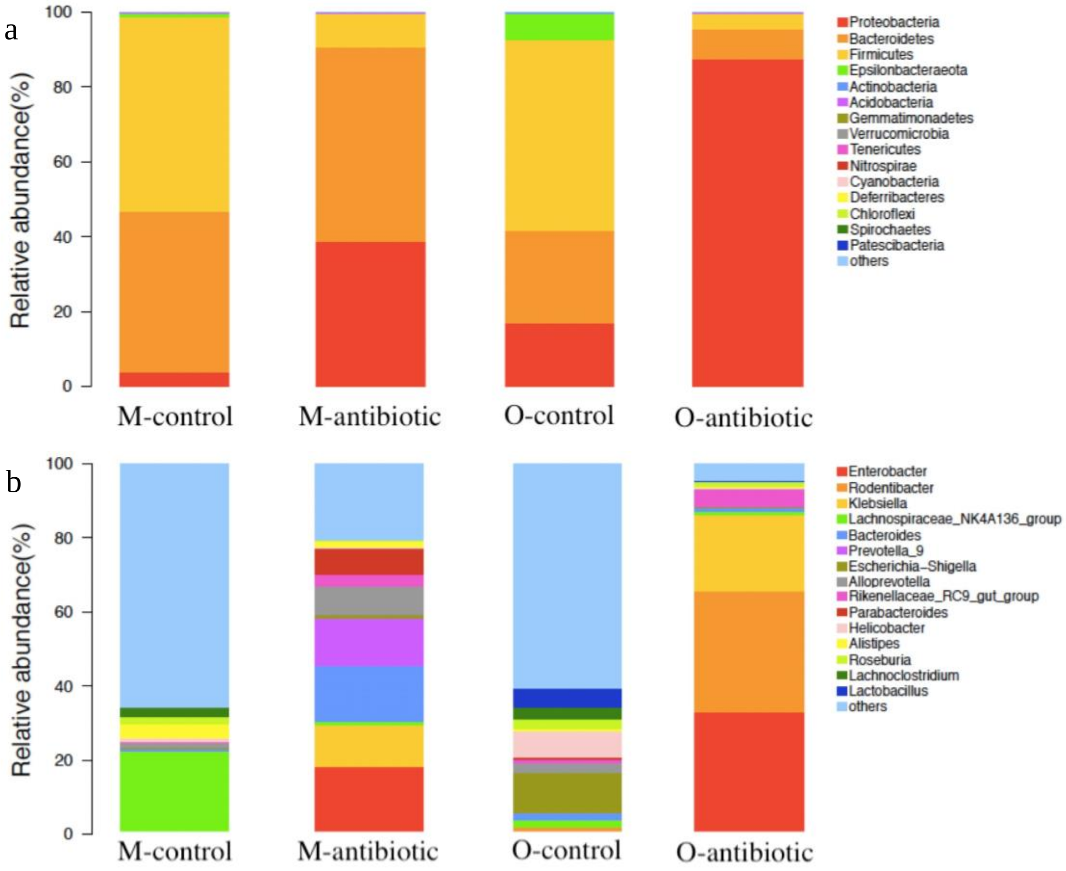

Figure S2. Relative abundance of top 15 phyla (a) and 15 genera (b) that were significantly different between mouse groups. M-antibiotics and M-control stand for antibiotics-treated mothers and control mothers, respectively, while M-antibiotics and M-control represent offspring born to antibiotics-treated mothers and control mothers, respectively.

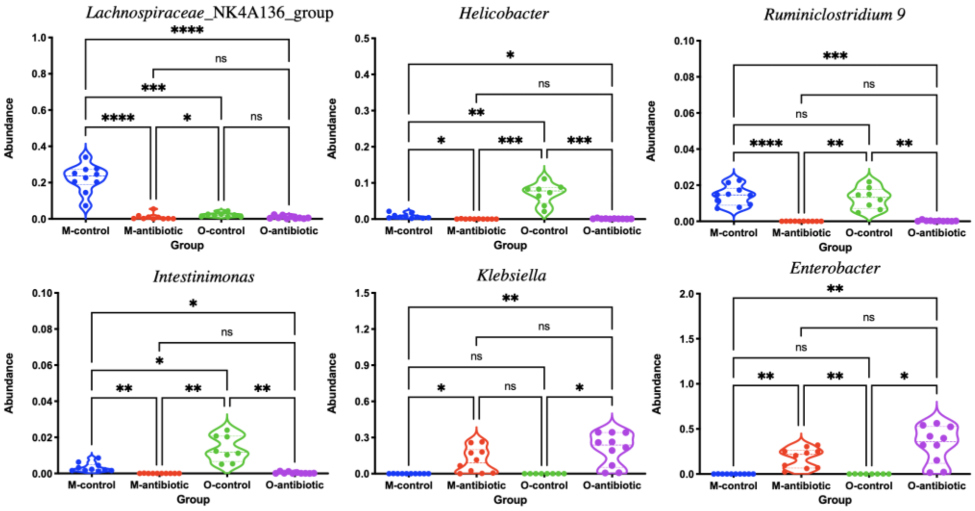

Figure S3. Relative abundance of six genera that were significantly different in the gut microbiota in the maternal and offspring samples. M-antibiotics and M-control stand for antibiotics-treated mothers and control mothers, respectively, while M-antibiotics and M-control represent offspring born to antibiotics-treated mothers and control mothers, respectively. Statistical significance: *, P < 0.05; **, P < 0.01; ***, P < 0.001; ns, not significant.
